# Photodynamic therapy for radiation maculopathy: A case report

**DOI:** 10.1097/MD.0000000000024888

**Published:** 2021-04-09

**Authors:** Taro Baba, Kazuyuki Hirooka, Yuki Yuasa, Noriko Ihara, Joji Takenaka, Hideaki Okumichi, Yoshiaki Kiuchi

**Affiliations:** Department of Ophthalmology and Visual Science, Hiroshima University Graduate School of Biomedical Sciences.

**Keywords:** photodynamic therapy, radiation maculopathy

## Abstract

**Rationale:**

The best treatment protocol for radiation maculopathy in children has not been determined. The purpose of this study was to determine the effect of photodynamic therapy (PDT) on radiation maculopathy.

**Patient concerns:**

An 11-year-old boy who was originally diagnosed with orbital rhabdomyosarcoma when he was 1 year old, in October 2008. The lesion improved after peripheral blood stem cell transplantation, chemotherapy and radiation therapy. A cataract was detected in his right eye in May 2011, and he underwent cataract surgery in July 2011. Continuous amblyopia training maintained his visual acuity in his right eye. In January 2017, his visual acuity was reduced and macular edema was detected with optical coherence tomography.

**Diagnoses:**

We diagnosed radiation maculopathy, from the history of radiation therapy, macular edema by optical coherence tomography, and hyperfluorescent site by fluorescein angiography.

**Interventions:**

We performed PDT in June 2017.

**Outcomes:**

Treatment with PDT improved macular edema and his visual acuity.

**Lessons:**

Radiation retinopathy is progressive disorder with poor prognosis. PDT could be considered to treat radiation maculopathy.

## 1 Introduction

Radiation retinopathy is a vascular disorder that develops after irradiation of the periorbital region.^[1,2]^ It manifests as ischemic and proliferative changes in the retina, leading to maculopathy.^[3]^ When ischemia occurs in the retina, vascular endothelial growth factor (VEGF) is released. The VEGF induces neovascularization and enhances vascular permeability, which leads to edema of the macular region. Because radiation retinopathy can progress to retinal degeneration, photocoagulation of the ischemic area is important to resolve the retinal ischemia.^[4]^

Photodynamic therapy (PDT) using verteporfin,^[4,5]^ sub-Tenon's triamcinolone acetonide injections^[4]^ and vitreal injections of anti-VEGF agents have been reported to be effective treatments for radiation maculopathy.^[4]^ However, the best treatment protocol for radiation maculopathy in children has not been determined.

We report the results of PDT in an 11-year-old boy who underwent radiation therapy for an orbital rhabdomyosarcoma when he was 1 year old, and later developed radiation maculopathy.

## 2 Case report

A 1-year-old boy was noted to have protrusion of his right eye and was diagnosed with the fetal type of orbital rhabdomyosarcoma in October 2008. The size of the mass was reduced by a peripheral blood stem cell transplantation, chemotherapy and radiation therapy with 50 gray units in the period from November 2008 to June 2009. He developed a cataract in the right eye in May 2011, and his visual acuity in that eye decreased to hand motion. He underwent phacoemulsification and aspiration, and intraocular lens implantation in the right eye in July 2011. There were no abnormal findings on fluorescein angiography, which was performed soon after the cataract surgery. His visual acuity after the cataract removal was 20/200 and, with amblyopia training, his visual acuity improved to 20/25. In May 2016, he underwent YAG-laser posterior capsulotomy and his visual acuity was maintained at 20/25.

**Figure 1:**
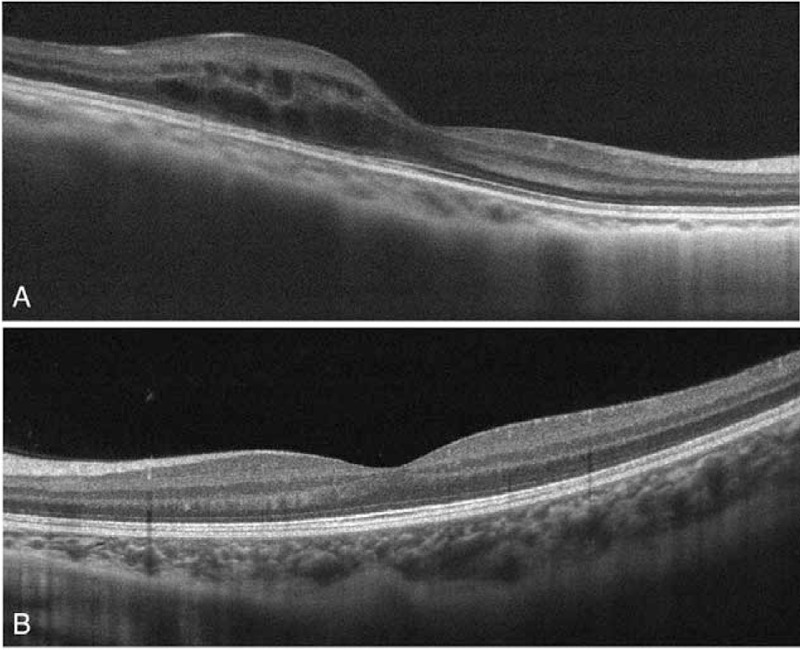
Optical coherence tomography showed macular edema in the outer plexiform layer of the right eye (A). The left eye was completely normal (B).

**Figure 2:**
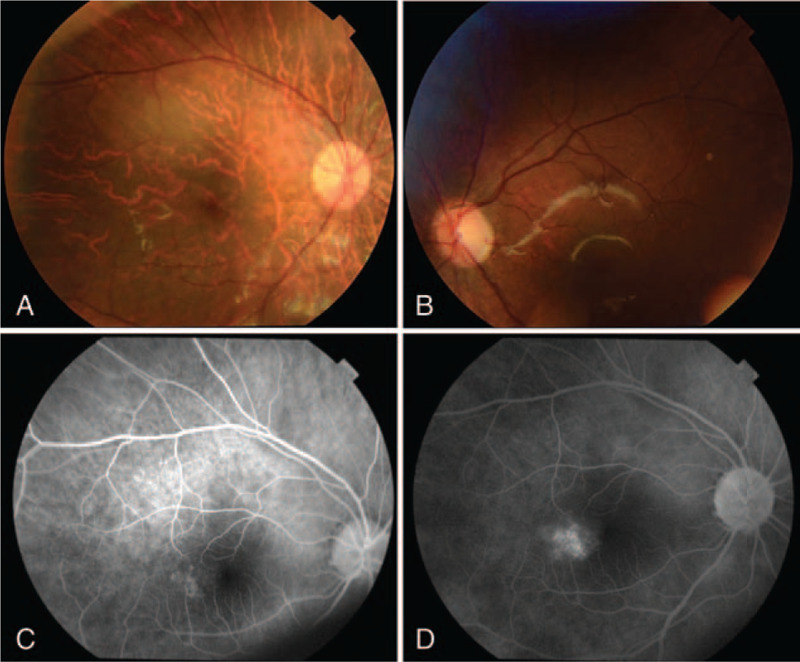
Fundus photographs. There were no soft exudates or retinal hemorrhages in the right eye (A). There are no abnormalities in the left eye (B). Fluorescein angiography (FA) showed that there was a hyperfluorescent site on the right side of the fovea in the right eye. The fluorescent site enlarged at the late phase of FA. Also note that there are no nonperfused areas. Early phase (C), late phase (D). We couldn’t measure time because he was crying during the examination.

At a regular examination in January 2017, his visual acuity was 20/60 in the right eye and 20/20 in the left eye. The intraocular pressure was 13 mm Hg in both eyes, as measured with noncontact tonometry. Slit-lamp microscopy showed superficial punctate keratopathy of the right eye. The left eye was clear. The bulbar conjunctiva was not injected in either eye. The anterior chamber of the right eye was deep and there were no signs of inflammation. The depth of the anterior chamber of the left eye was normal and the lens of the left eye was clear. Examination of the fundus by indirect ophthalmoscopy showed neither a retinal detachment nor retinal hemorrhages in either eye. Optical coherence tomography (OCT) showed macular edema in the right eye (Fig. 1). There were no soft exudates or retinal hemorrhages (Fig. 2). Fluorescein angiography showed a hyperfluorescent site on the right side of the fovea of the right eye, but there were no nonperfused areas (Fig. 2). The macular edema was considered to be due to the prior radiation therapy.

**Figure 3:**
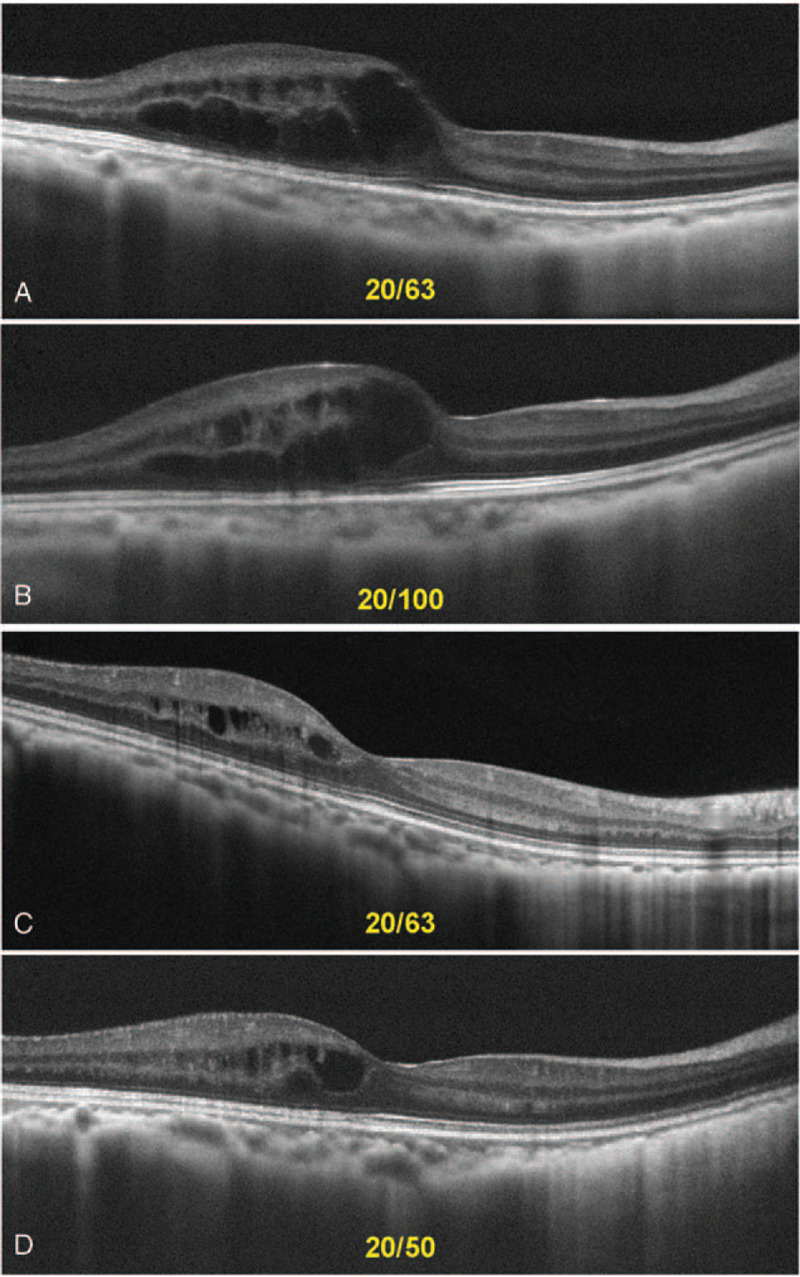
Visual acuity improved after the photodynamic therapy (PDT), and the macular edema decreased. Visual acuity values are shown on the figure panels for the following time points: before PDT (A), and after PDT at: 12 months (B), 24 months (C), 36 months (D).

**Figure 4:**
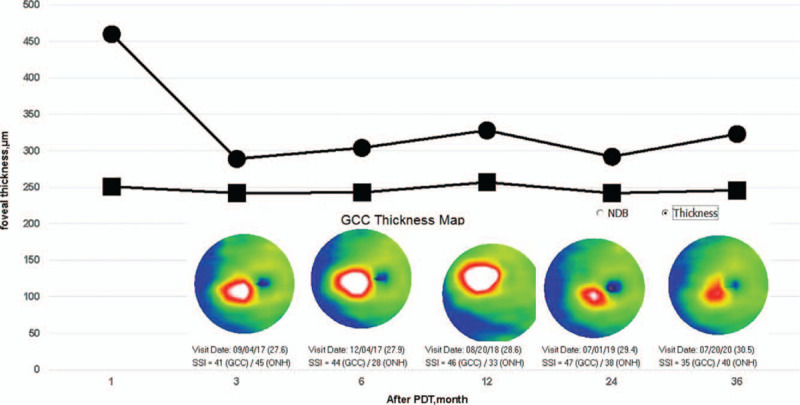
Optical coherence tomography maps of the right retina. The foveal thickness decreased from 1 month to 3 months after treatment but then gradually increased. Ganglion Cell Complex (GCC) Thickness Map are shown on the figure panels for the following time points. It can be seen that GCC is gradually decreasing after the PDT. 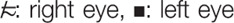.

As mentioned above, different types of treatment have been used for radiation maculopathy, including a sub-Tenon's injection of triamcinolone acetonide, intravitreal anti-VEGF agents and PDT using verteporfin.^[3,4]^ We explained these options to his parents, and they chose PDT because the anti-VEGF treatment would have required repeated intravitreal injections. However, PDT for this type of disorder had not been approved in Japan at that time. After receiving approval for PDT from our Ethics Committee, we performed low-emission energy PDT, in June 2017 with irradiation energy of 25 J/cm^2^ for 45 seconds. The patient's visual acuity improved after the treatment, and the macular edema decreased after the PDT (Fig. 3). OCT showed that the foveal thickness decreased between 1 month and 3 months after the PDT but then gradually increased (Fig. 4). However, in the long run the Ganglion Cell Complex is gradually decreasing after the PDT (Fig. 4).

At a follow-up visit in July 2020, his visual acuity was 20/50 in the right eye and 20/20 in the left eye. OCT showed macular edema in the outer plexiform layer of the right eye, but the left eye was completely normal.

## 3 Discussion

The development of radiation retinopathy is dependent on the dose of radiation, and individuals exposed to radiation of 45 to 55  gray are considered to be susceptible.^[6]^ Radiation retinopathy generally occurs between 6 months and 3 years after periorbital radiation is performed.^[7]^ The pathological changes are triggered by damage to the retinal blood vessels, which develops late and progresses slowly.^[1]^

In radiation maculopathy, the retina is in an ischemic state and VEGF is expressed. These changes increase the permeability of the retinal blood vessels. In addition, the radiation affects the retinal pigment epithelium cells and choroidal blood vessels. It is believed that intravitreal anti-VEGF drugs may be effective in treating radiation maculopathy.^[2]^ Several studies have reported on the effects of anti-VEGF therapy on radiation maculopathy. Their results suggest that, although intravitreal anti-VEGF drugs reduce the macular edema, the effect is temporary. In many cases, repeated intravitreal injections are required to maintain the effect and the visual acuity improvement is not significant.^[4]^

The mechanism by which PDT affects radiation maculopathy is not well-understood. Although grid retinal photocoagulation is effective for the treatment of macular edema, its mechanism of action is believed to be enhancement of the oxygen permeability of the retinal pigment epithelium cells, which then allows more oxygen to flow from the choroid to the outer retina. In addition, cytokines are released from the retinal pigment epithelium cells and their surrounding tissues in response to PDT, which reduces the edema.^[5]^ However, PDT occludes blood vessels that have taken in bisdyne, and may also suppress the permeability of the blood vessels.^[8]^

Three months after the PDT, the patient's visual acuity temporarily improved and the macular edema also improved, but it did not completely disappear. The visual acuity varied with the presence of superficial punctate keratopathy, which we suggest was associated with the radiation injury. Factors beyond visual acuity should be explored in the evaluation of the effects of PDT. The foveal thickness decreased until 3 months after the PDT and then increased again. The effects of PDT, thus, were temporary, as is the case with other treatments. However, in the long run, Ganglion Cell Complex is declining, so PDT is considered an effective treatment. It has also been reported that PDT can cause damage to choroidal vessels, so repeat use of PDT is discouraged.

In conclusion, radiation maculopathy is a progressive disorder with poor prognosis, so it should be prevented. Our treatment with PDT improved macular edema and improved his visual acuity. However, this was a single case, and additional reports of radiation maculopathy treated with PDT are needed to prove that PDT is a successful treatment protocol.

## Acknowledgment

We thank Duco I. Hamasaki, Ph.D., from Miami University for proofreading. We thank Claire Barnes, PhD, from Edanz Group (www.edanzediting.com/ac) for editing a draft of this manuscript.

## Author contributions

**Resources**: Yoshiaki Kiuchi.

**Supervision**: Yoshiaki Kiuchi.

**Visualization**: Taro Baba.

**Writing – original draft**: Taro Baba, Yuki Yuasa, Hideaki Okumichi, Yoshiaki Kiuchi.

**Writing – review & editing**: Kazuyuki Hirooka, Yoshiaki Kiuchi.
